# Resveratrol enhances pluripotency of mouse embryonic stem cells by activating AMPK/Ulk1 pathway

**DOI:** 10.1038/s41420-019-0137-y

**Published:** 2019-02-04

**Authors:** Irina I. Suvorova, Aleksandra R. Knyazeva, Alexey V. Petukhov, Nicolay D. Aksenov, Valery A. Pospelov

**Affiliations:** 1 Institute of Cytology, Russian Academy of Sciences, St-Petersburg, Russian Federation; 2 Saint-Petersburg State University, St-Petersburg, Russian Federation; 3 Institute of Hematology, Almazov Federal North-West Medical Research Centre, Saint-Petersburg, Russian Federation

## Abstract

Resveratrol, a natural polyphenolic compound, shows many beneficial effects in various animal models. It increases efficiency of somatic cell reprograming into iPSCs and contributes to cell differentiation. Here, we studied the effect of resveratrol on proliferation and pluripotency of mouse embryonic stem cells (mESCs). Our results demonstrate that resveratrol induces autophagy in mESCs that is provided by the activation of the AMPK/Ulk1 pathway and the concomitant suppression of the activity of the mTORC1 signaling cascade. These events correlate with the enhanced expression of pluripotency markers Oct3/4, Sox2, Nanog, Klf4, SSEA-1 and alkaline phosphatase. Pluripotency is retained under resveratrol-caused retardation of cell proliferation. Given that the Ulk1 overexpression enhances pluripotency of mESCs, the available data evidence that mTOR/Ulk1/AMPK-autophagy network provides the resveratrol-mediated regulation of mESC pluripotency. The capability of resveratrol to support the mESC pluripotency provides a new approach for developing a defined medium for ESC culturing as well as for better understanding signaling events that govern self-renewal and pluripotency.

## Introduction

Embryonic stem cells (ESCs) are pluripotent cells and therefore attract much attention due to the potential use in tissue replacement therapy. Since pluripotency is a transient cell state in vivo, it remains unclear how sustained propagation of ESCs can be maintained in vitro. Hence, it is critical to develop the most optimal conditions for ESC culturing. Serum-based cultures of ESCs produce heterogeneous cell populations after a long-term passaging in vitro as evidenced by morphological changes, reduced self-renewal and spontaneous differentiation. Therefore, the maintenance of stable pluripotent stem cells in the long-term culture is one of the most important tasks of cell therapy. Recently, a defined media supplemented with two inhibitors of MEK and GSK3 with LIF (2i/LIF) to maintain mouse embryonic stem cells (mESCs) in a naive ground state was reported^1^. However, prolonged cultivation of male mESCs in such cocktail results in irreversible epigenetic and genomic changes that impair their developmental potential^2^. Several protocols have been developed for establishment of naive human ESC cultures that are based on 2i/LIF supplemented with additional components and/or with additional genetic manipulations^1–7^. The reported cocktails used to induce human naive pluripotency likely cause a spectrum of pluripotent states^8^. Hence, the search of agents that could be included in the mouse and human ESC protocols is to be continued. Using small molecules instead of genetic manipulations is more preferable, since their action is reversible and adjustable. Resveratrol (3,4,5-trihydroxy-trans-stilbene) is a polyphenolic phytoalexin widely presented in some plants^9^. Accumulating reports have shown that resveratrol can prevent or slow down the progression of a wide variety of diseases, including cancer, cardiovascular diseases and Alzheimer’s disease as well as enhance stress resistance and extend the lifespan of various organisms from yeast to vertebrates^10^. The beneficial effects of resveratrol on a large number of cellular processes allowed us to assume that this promising compound can also be useful in the positive regulation of the fundamental properties of ESCs—self-renewal and pluripotency. In favor of this assumption, there is an evidence that supplementation of resveratrol has beneficial effect on porcine and cow in vitro fertilization and subsequent embryonic development^11,12^. The addition of resveratrol to the medium for cultivation of pig oocytes allows to obtain more viable blastocysts and efficiently isolate ESCs from them^11^. Several studies have reported the effects of resveratrol on mESC differentiation, pluripotency and cell reprogramming^13–16^. However, there is the complexity of determining the main mechanisms of resveratrol action due to the large number of its targets. Therefore, the precise mechanisms of resveratrol effects on pluripotency and self-renewal remain to be elucidated. Here, we demonstrate a novel mechanism of resveratrol action on undifferentiated mESCs. Our results show that resveratrol maintains mESC pluripotency due to autophagy induction through activation of the AMPK/Ulk1 pathway and downregulation of mammalian target of rapamycin complex 1 (mTORC1). In addition, by overexpressing the Ulk1-bearing construct under doxycyclin regulation in mESCs, we show that the AMPK/Ulk1 (adenosine monophosphate-activated protein kinase/Unc-51 like autophagy activating kinase 1) signaling augments the expressions of pluripotency factors Oct3/4, Sox2, Klf4 and Nanog that maintain mESCs in undifferentiated state.

## Results

### Resveratrol induces S-phase cell cycle delay in mESCs

**Fig. 1:**
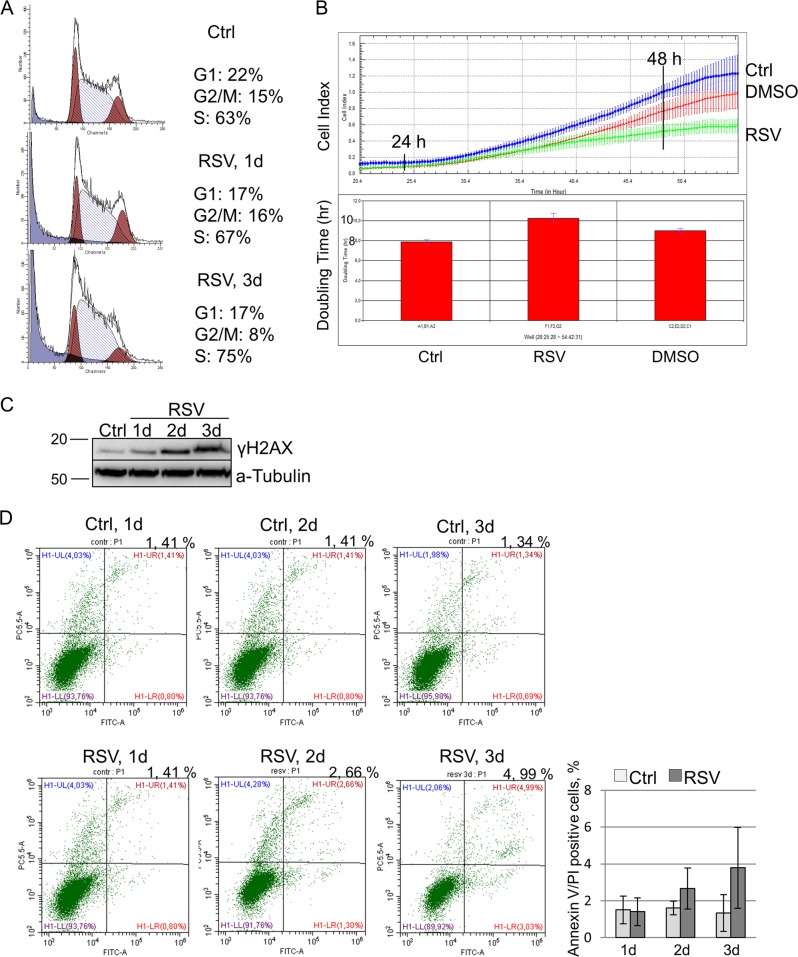
Resveratrol induces S-phase cell cycle delay in mouse embryonic stem cells (mESCs). **a** Fluorescence-activated cell sorting (FACS) analysis of cell cycle phase distribution of mESCs untreated and treated with 10 µM of resveratrol for 1 and 3 days. **b** Top panel: cell index curves of untreated and resveratrol-treated (10 µM) mESCs were generated using the xCELLigence RTCA DP system. The proliferation rates were registered every 15 min for 55 h; error bars correspond as means ± SEM (*n* = 4). Bottom panel: the values were obtained at the whole proliferation curve at 0–55 h. **c** Western blot of total lysates from control mESCs and mESCs treated with resveratrol (10 µM) for the indicated periods of time; antibodies against H2AX (Ser139) and α-Tubulin for endogenous control were used. The representative of experiments repeated at least three times is shown. **d** Left panel: flow cytometric analysis of apoptotic cells using Annexin V surface staining and propidium iodide (PI). Shown are dot plots of the fluorescein isothiocyanate-conjugated Annexin V versus PI staining for mESCs untreated and treated with resveratrol for the indicated periods of time. Right panel: representation of flow cytometry results from the left panel as diagram; error bars correspond to the SEM calculated for three replicates

Following resveratrol treatment (RSV), mESCs accumulate in the S phase of cell cycle (Fig. 1a). This increase is relatively small (ca. 12%) compared to control mESCs (63%) but because mESCs have high proliferation rate with predominant distribution in the S phase of cell cycle, the observed S-phase increase can be considered as substantial. Accumulation of resveratrol-treated mESCs in the S phase suggests a temporary S-phase delay and therefore an increase of the cell-doubling time. To clarify this issue, we performed a real-time comparative analysis of proliferation rate of mESCs for 60 h using the xCELLigence real-time cell analysis, dual purpose (RTCA DP) system that enables us to analyze the detailed cell proliferation dynamics. The obtained growth curves show that resveratrol-treated mESCs divide slower than the untreated cells (Fig. 1b, top panel). Correspondingly, the cell-doubling time after resveratrol treatment slightly exceeds 10 h as compared with 9.0 h in the untreated control (Fig. 1b, bottom panel). Using antibodies against phosphorylated H2AX histone (γH2AX Ser139), we checked whether RSV-induced modulation of DNA replication is recognized as a replicative stress (Fig. 1c). It is well known that γH2AX foci are the markers of double-stranded DNA breaks, replication fork collapse and replicative stress caused by unscheduled replication. According to data obtained, resveratrol induced a gradual γH2AX accumulation after treatment of mESCs for 1, 3 and 5 days. These results show that resveratrol causes an unscheduled DNA replication followed by a temporary S-phase delay that is recognized by the cell as a replicative stress with the concomitant activation of the DNA damage repair machinery. To assess whether the S-phase delay is detrimental for mESCs, we performed Annexin V-fluorescein isothiocyanate (FITC) staining to detect apoptosis in cells. Exposure of mESCs to 10 μM of resveratrol for 1–3 days showed no difference on cell viability compared to control (Fig. 1d). Thus, resveratrol treatment decreased mESC proliferation by inducing a S-phase delay that is not accompanied by apoptosis induction.

### Resveratrol has positive effect on mitochondrial metabolism in mESCs

**Fig. 2:**
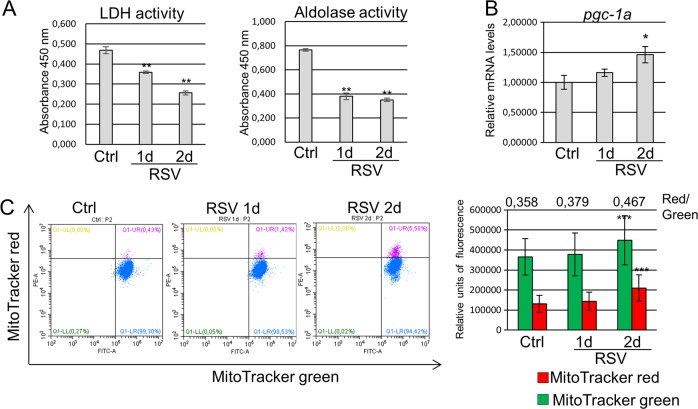
Resveratrol accelerates mitochondrial functions in mouse embryonic stem cells (mESCs). **a** mESCs were treated with 10 µM resveratrol for 1 and 2 days. The lactate dehydrogenase (LDH) activity (left panel) and the aldolase activity (right panel) in mESC lysates were spectrophotometrically monitored at 450 nM and normalized by the total protein concentration in cell extracts. Error bars correspond to the SEM calculated for three replicates; **p < 0.001. **b** qRT-PCR analysis of gene expression in control mESCs and mESCs treated with resveratrol (10 µM) for the indicated periods of time. The data are normalized to *β-actin* expression. Error bars correspond to the SEM calculated for four replicates; *p < 0.05. **c** Left panel: flow cytometry analysis of untreated and resveratrol-treated mESCs stained with MitoTracker green and MitoTracker red.  Fluorescence intensity was measured on a flow cytometer in both the red (PE-A) and green (fluorescein isothiocyanate (FITC)-A) channels. Right panel: representation of flow cytometry results from left panel as diagrams; the number of mitochondria were calculated as the ratio MitoRed/MitoGreen; data are represented as mean ± rSD (*n* = 10,000); ***p < 0.0001. rSD relative standard deviation

Thus, to some extent resveratrol inhibits cell proliferation which is accompanied by S-phase delay (Fig. 1). Such a decrease of mESC proliferation can be due to a modulation of metabolic activity, in particular glycolysis. Indeed, the enzymatic activity of key glycolysis proteins lactate dehydrogenase (LDH) and aldolase in resveratrol-treated cells has been shown to decrease, implying that resveratrol down-regulates the activity of LDH and aldolase in mESCs (Fig. 2a).

Resveratrol is reported to affect the mitochondrial activity^15,17^. Peroxisome proliferator-activated receptor-γ coactivator-1α (PGC-1α) is a transcriptional coregulator of genes involved in energy metabolism. A strong correlation between PGC-1α expression and mitochondrial biogenesis was early established^18^. As presented in Fig. 2b, transcript level of *pgc-1a* gene was significantly increased after 2 days of resveratrol treatment. To determine whether resveratrol influences the function of mitochondria in mESCs, we applied the fluorescence markers MitoTracker green (a mitochondria selective membrane potential-independent probe) and MitoTracker Red (a mitochondria selective membrane potential-dependent probe). According to data obtained, statistically significant enhanced MitoTracker green staining is detected after 2 days of resveratrol treatment of mESCs that correlates with upregulation of *pgc1*-*a* gene expression and indicates mitochondrial biogenesis (Fig. 2c). The ratio of the red to green fluorescence intensity increases and confirms the production of active mitochondria in resveratrol-treated cells. Thus, the dependency of energy production on glycolysis decreases in resveratrol-treated mESCs, while the mitochondrial metabolism increases.

### Resveratrol enhances mESC pluripotency

**Fig. 3:**
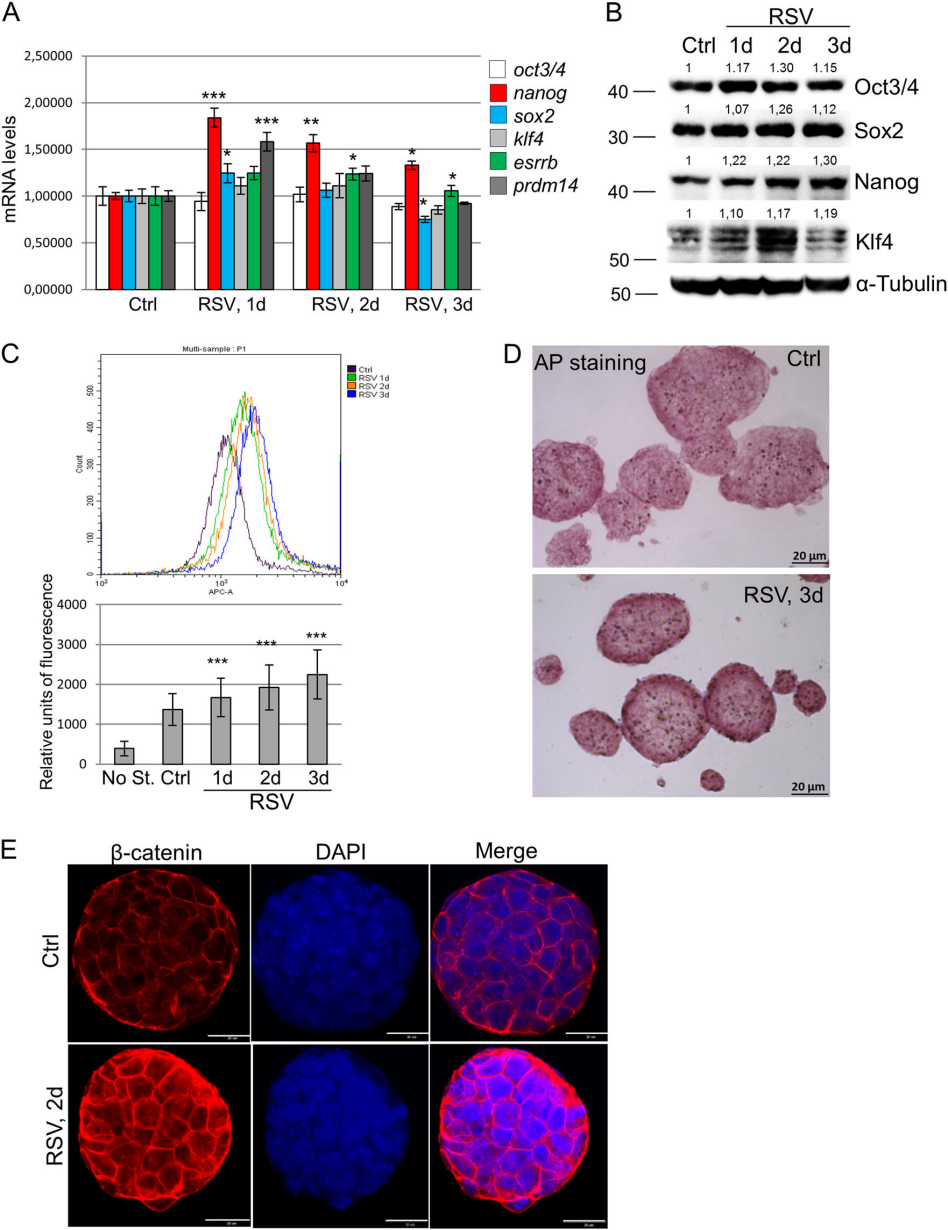
Resveratrol enhances mouse embryonic stem cell (mESC) pluripotency. **a**. qRT-PCR analysis of gene expression in control mESCs and mESCs treated with resveratrol (10 µM) for the indicated periods of time. The data are normalized to *β-actin* expression. Error bars correspond to the SEM calculated for four replicates; *p < 0.05, **p < 0.001, ***p < 0.0001. **b** Top panel: western blot of total lysates from control mESCs and mESCs treated with resveratrol (10 µM) for the indicated periods of time; antibodies against Oct3/4, Nanog, Sox2, Klf4 and α-Tubulin for endogenous control were used; the data are normalized to α-Tubulin. The representative of experiments repeated at least three times is shown. **c** Top panel: flow cytometry analysis of mESCs exposed to SSEA-1 antibody staining; mESCs were treated with 10 µM resveratrol for the indicated periods of time. The cells were incubated with secondary antibodies AlexaFlour-568-conjugated goat anti-mouse F(ab′)2 fragment and observed with flow cytometer in APC-A channel. Bottom panel: representation of flow cytometry results as diagram; data are represented as mean ± rSD (*n* = 10,000). rSD relative standard deviation; ***p < 0.0001. **d** Alkaline phosphatase (AP) staining of untreated mESCs and mESCs treated with resveratrol (10 µM) for 3 days. **e** Resveratrol results in accumulation of β-catenin in mESCs. Immunofluorescent staining of untreated and resveratrol-treated mESCs (2 days) with antibody to β-catenin (red). Nuclei were stained with 4′,6-diamidino-2-phenylindole (DAPI; blue). Scale bar 20 µm

Pluripotency of ESCs is maintained by orchestrated network of regulatory pluripotency-associated transcription factors such as Oct3/4, Sox2, Nanog, Klf4 as well as ESRRB and PRDM14. As presented in Fig. 3a, resveratrol induces a significant increase of *nanog* gene transcription at the specified time-course intervals. Less significant (but still noticeable) upregulation of the *sox2, prdm14* and *esrrb* gene expression in mESCs after resveratrol treatment for 1–3 days has been detected (Fig. 3a). However, *oct3/4* and *klf4* messenger RNA (mRNA) transcripts remain almost on the same levels after resveratrol. Importantly, western blot data show the elevated expression levels of Oct3/4, Sox2, Nanog and Klf4 proteins in resveratrol-treated cells, whereas significant Nanog accumulation correlates with the corresponding up-regulation of *nanog* mRNA transcripts (Fig. 3b). Of note, ESCs maintain an optimal level of pluripotency proteins and any changes thereof  are  considered as significant. To confirm observed resveratrol effect on pluripotency, we examined by flow cytometry the expression levels of a cell surface marker, stage specific embryonic antigen-1 (SSEA-1), a sensitive and specific marker for undifferentiated mESCs (Fig. 3c). As expected, SSEA-1 expression becomes substantially higher in resveratrol-treated cells than in the untreated control cells. Alkaline phosphatase (AP) expression is another marker of undifferentiated state of ESCs . The AP-stained cells display bright staining that confirms retention of pluripotency of resveratrol-treated cells (Fig. 3d). Morphologically, resveratrol-treated mESCs exhibited more tightly packed colonies with mainly smooth boundaries suggesting an increase of cell adhesive properties. This observation is further confirmed by β-catenin staining, which participates in tight intercellular adhesions mediated through cadherin–catenin contacts. As visualized by confocal immunofluorescence, more intensive membrane staining of β-catenin is detected in resveratrol-treated cells relative to untreated mESCs (Fig. 3e). All together, it gives an evidence for a conclusion that resveratrol not only maintains but also enhances pluripotency of mESCs.

### Resveratrol maintains pluripotency through AMPK/Ulk1-dependent autophagy in mESCs

**Fig. 4:**
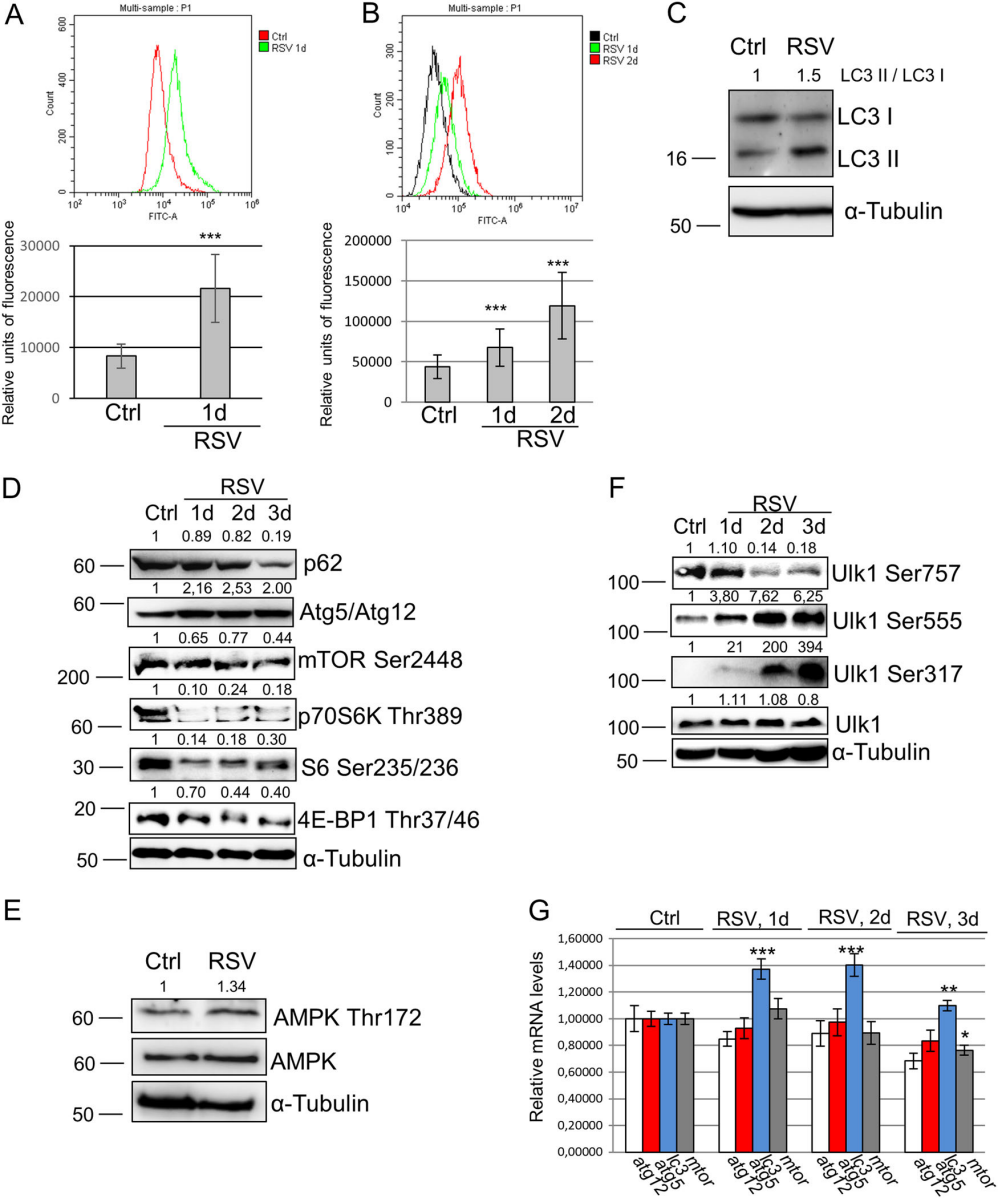
Resveratrol activates AMPK/Ulk1-dependent autophagy in mouse embryonic stem cells (mESCs). **a** Upper panel: flow cytometry of fluorescent probe based on monodansylcadaverine (MDC); bottom panel: representation of flow cytometry results as diagram; data are represented as mean ± rSD (*n* = 10,000). rSD relative standard deviation; ***p < 0.0001. mESCs were treated with 10 µM resveratrol and chloroquine (10 µM) for 1 day. **b** Upper panel: flow cytometry analysis of LysoTracker green staining of mESCs treated with 10 µM resveratrol for the indicated periods of time. Bottom panel: representation of flow cytometry results as diagram; data are represented as mean ± rSD (*n* = 10,000); ***p < 0.0001. **c** Western blot of total lysates from control mESCs and mESCs treated with resveratrol (10 µM) for 1 day using antibodies against LC3; quantitative assessment of the ratio LC3-II/LC3-I, this ratio is normalized to α-Tubulin, the ratio obtained represents the autophagic rate. The representative of experiments repeated at least three times is shown. **d** Western blot of total lysates from control mESCs and mESCs treated with resveratrol (10 µM) for the indicated periods of time; the data are normalized to α-Tubulin. The representative of experiments repeated at least three times is shown. **e** Western blot of total lysates from control mESCs and mESCs treated with resveratrol (10 µM) for 1 day; quantitative assessment of the ratio AMPK Thr172/AMPK, this ratio is normalized to α-Tubulin. The representative of experiments repeated at least three times is shown. **f** Western blot of total lysates from control mESCs and mESCs treated with resveratrol (10 µM) for the indicated periods of time; the data are normalized to α-Tubulin. The representative of experiments repeated at least three times is shown. The representative of experiments repeated at least three times is shown. **g** qRT-PCR analysis of selected genes in control mESCs and mESCs treated with resveratrol (10 µM) for the indicated periods of time. The data are normalized to *β-actin *expression. Error bars correspond to the SEM calculated for four replicates; *p < 0.05, **p < 0.001, ***p < 0.0001. AMPK/Ulk1 adenosine monophosphate-activated protein kinase/Unc-51 like autophagy activating kinase 1

As shown above, resveratrol induces production of active mitochondria in mESCs. This can be explained by mitophagy-driven mitochondrial rejuvenation. Thus, we assessed autophagy in resveratrol-treated mESCs. Cyto-ID test is a convenient method for evaluation of autophagy induction in cells based on monodansylcadaverine (MDC) fluorescent dye. Cyto-ID staining showed that the fluorescent density of resveratrol-treated cells for 1 day were higher than in untreated mESCs (Fig. 4a). Consistent with the induced MDC-dependent fluorescence, LysoTracker green staining of resveratrol-treated mESCs confirms the lysosome accumulation (Fig. 4b). Activation of autophagy is accompanied by a conversion of LC3-I to a autophagosome membrane-bound LC3-II form, thus considering the membrane-bound LC3-II as an indicator of autophagolysosome formation (Fig. 4c). Moreover, we detected that the Atg5/Atg12 dimer, a marker of ongoing autophagy, also accumulates in resveratrol-treated cells, while p62/SQSTM1 adaptor protein level decreases after resveratrol treatment that is consistent with active autophagy (Fig. 4d).

To get more insight into the mechanisms of resveratrol-induced autophagy, we assessed the mTORC1 targets. The activation of mTORC1 signaling negatively regulates autophagy and, in turn, suppression of mTORC1 leads to autophagy flux. Under resveratrol treatment of mESCs, mTOR kinase activity was downregulated as tested by a decrease of mTOR phosphorylation at Ser2448 (Fig. 4d). We checked direct downstream targets of mTORC1 and showed a decrease of phosphorylation of p70/80S6 at Thr389, pS6 at Ser235/236 and p4EBP1 at Thr37/46, thereby implying that resveratrol down-regulates the mTORC1 pathway in mESCs (Fig. 4d). In turn, mTOR negatively regulates autophagy through inhibitory phosphorylation of Ulk1 at Ser757, while inhibition of mTOR decreases the inhibitory phosphorylation level of Ulk1 and increases autophagy flux. AMPK upregulates autophagy in response to energy depletion through direct phosphorylation of Ulk1 at Ser555 and Ser317. Conversely, mTOR phosphorylates Ulk1 at Ser757 and disrupts the interaction between Ulk1 and AMPK. According to western blot data, AMPK is activated on Thr172 phosphorylation that correlates with Ulk1 phosphorylation on Ser555 and Ser317 in resveratrol-treated cells (Fig. 4e, f). We checked the expression levels of *atg5*, *atg12*, *lc3* and *mtor* genes (Fig. 4g): there is an increase of only *lc3* mRNA transcript level in mESCs treated with resveratrol for 1, 2 and 3 days. The expression of *mtor* gene was reduced in resveratrol-treated cells that is consistent with western blot results. Together, we conclude that resveratrol induces autophagy through downregulation of the mTORC1/Ulk1 pathway.

**Fig. 5:**
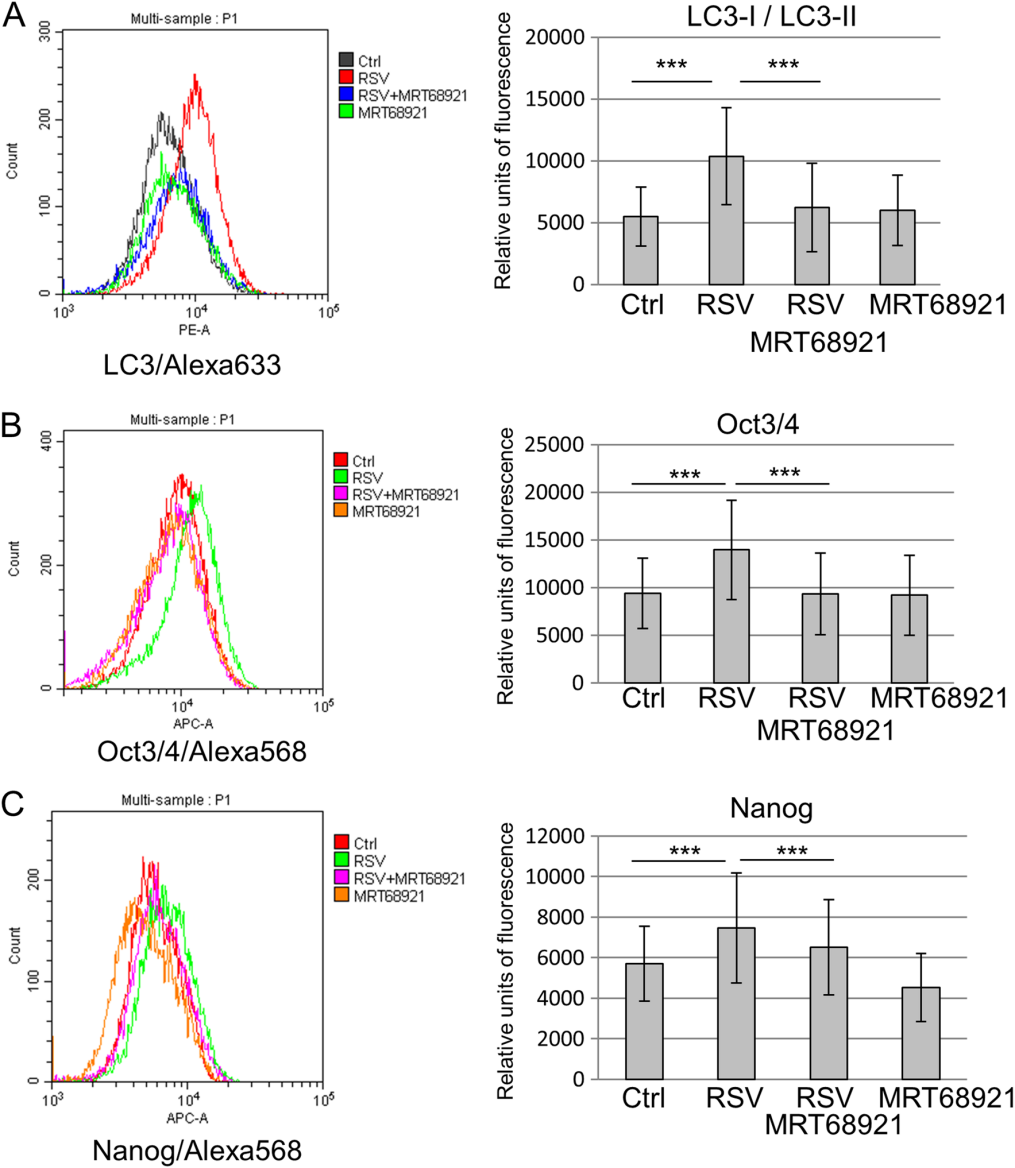
**Resveratrol maintains pluripotency through AMPK/Ulk1-dependent autophagy in mouse embryonic stem cells (mESCs)**. **a, b, c** Left panel: dual flow cytometric analysis of mESCs untreated and treated with resveratrol alone, in combination of resveratrol and MRT68921, and treated with only MRT68921 for 2 days. Cells were exposed to antibodies against Lc3, Oct3/4 and Nanog and further incubated with secondary antibodies AlexaFlour-568/633-conjugated goat anti-mouse/rabbit F(ab′)2 fragment. The cells were observed with flow cytometer in (PE-A) or (APC-A) channels. Right panel: cytometry histograms. Data are represented as mean ± rSD (*n* = 10,000). rSD relative standard deviation; ***p < 0.0001. The representative of experiments repeated at least three times is shown. AMPK/Ulk1 adenosine monophosphate-activated protein kinase/Unc-51 like autophagy activating kinase 1

Given that AMPK/Ulk1-dependent autophagy plays a key role in pluripotency regulation of resveratrol-treated mESCs, we used an inhibitor of Ulk1 activity MRT68921 and respectively Ulk1-dependent autophagy (Fig. 5). The flow cytometry as a quantitative and sensitive method capable of detecting even slight changes in pluripotency marker expression has been chosen. According to the data obtained, resveratrol induces LC3 protein accumulation abrogated by Ulk1 inhibition, thus evidencing for Ulk1-dependent inhibition of autophagy. In addition, resveratrol-induced increment of Oct4 and Nanog expression was also abrogated by Ulk1 inhibition. Therefore, resveratrol maintains the undifferentiated state of mESCs in vitro through AMPK/Ulk1-dependent autophagy activation.

### The Ulk1 activation stimulates AMPK/Ulk1 signaling pathway that is accompanied by enhanced pluripotency of mESCs

**Fig. 6:**
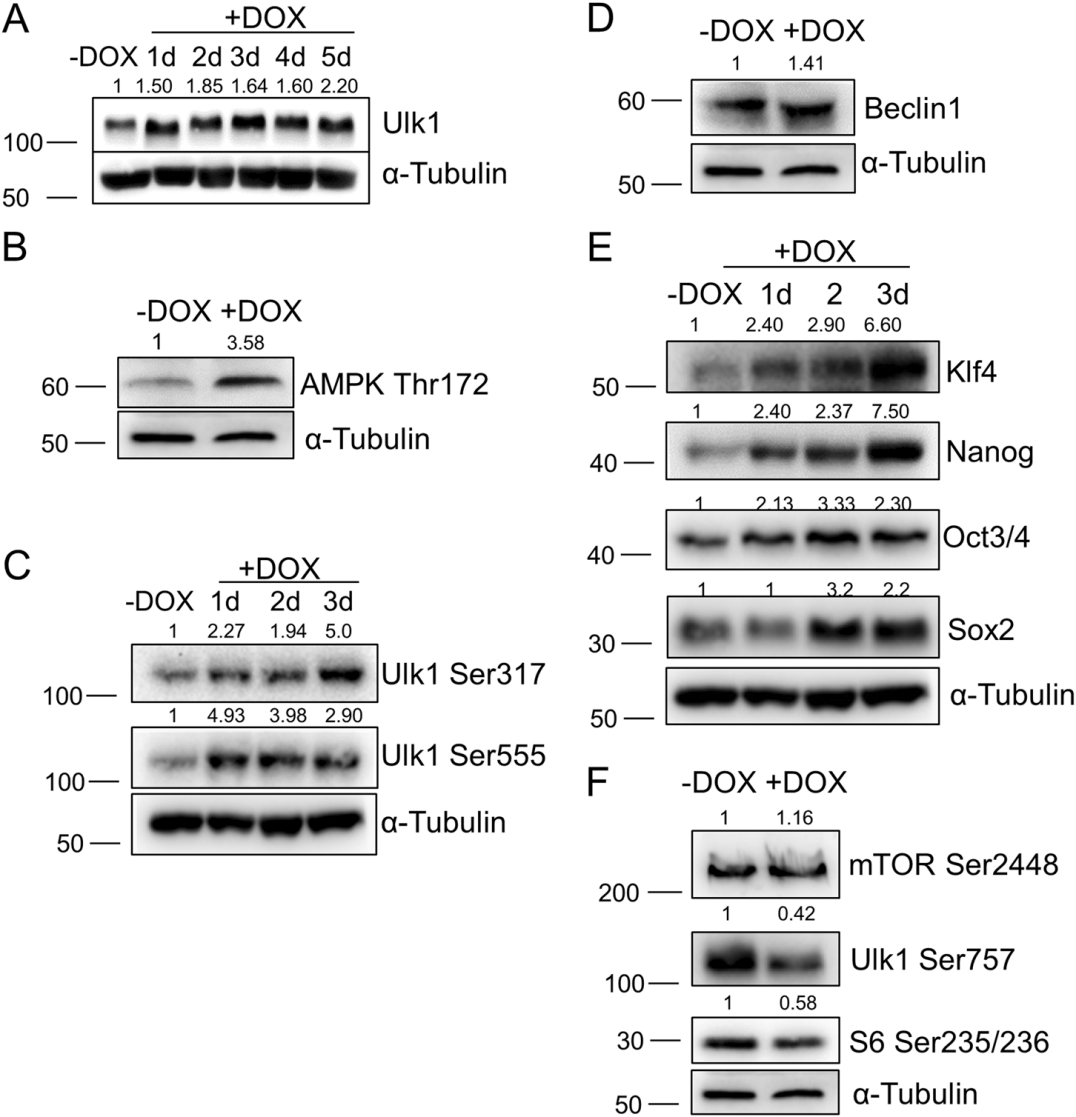
The Ulk1 activation induces AMPK/Ulk1 pathway activation that is accompanied with enhanced pluripotency of mouse embryonic stem cells (mESCs). Western blot of mESCs untreated and treated with doxycycline (5 µg/ml) from 1 to 5 days (**a**); for 1 day (**b**); from 1 to 3 days (**c**); for 1 day (**d**); from 1 to 3 days (**e**); and for 1 day (**f**). The data are normalized to α-Tubulin expression. The representative of experiments repeated at least three times is shown. AMPK/Ulk1 adenosine monophosphate-activated protein kinase/Unc-51 like autophagy activating kinase 1

We aimed to find out whether modulation of only the Ulk1 pathway is sufficient to affect pluripotency in mESCs. As an approach to assess this issue, we established a stable mESC line with doxycycline-inducible *ulk1* gene activation using CRISPRa technology. As shown at Fig. 6a, doxycycline triggers a significant accumulation of the steady level of Ulk1 protein in mESCs. A particular interest was to study the posttranslational phosphorylation profile of Ulk1 carried out by either AMPK or mTOR kinases in the mESCs after doxycycline treatment. Doxycycline-inducible Ulk1 protein is phosphorylated by AMPK at Ser555 and Ser317 residues, while phosphorylation of Ulk1 at Ser757, a mTOR-dependent site, decreased (Fig. 6b, c, f). Thus, autophagy signals likely going via Ulk1 negatively phosphorylate Ulk1 at Ser757 by mTOR to inhibit autophagy (Fig. 6f). Interestingly, AMPK becomes phosphorylated at Thr172 (activation) in mESCs after doxycycline-induced Ulk1 overexpression probably through feedback loops within mTOR/AMPK/Ulk1 network (Fig. 6b)^19^. Beclin-1 is another target of Ulk1 kinase under conditions of amino acid withdrawal^20^. Beclin-1 accumulates upon *ulk1* activation by doxycycline that promotes membrane nucleation of phagophores (Fig. 6d). Next, we studied the effect of AMPK/Ulk1-dependent autophagy on pluripotency and self-renewal of mESCs in details. As shown in Fig. 6e, an elevated expression of Ulk1 protein is accompanied by accumulation of pluripotency factors Oct3/4, Nanog, Sox2 and Klf4 in mESCs cultured for 1, 2 and 3 days in the presence of doxycycline (DOX, Fig. 6e). Although there is no decrease in mTOR kinase phosphorylation at Ser2448 (Fig. 6f), one can see that Ulk1 activation in DOX+mESCs interferes with mTOR signaling and negatively regulates S6 activity, a target of mTOR, as tested by its phosphorylation at Ser235/236 (Fig. 6f). Thus, serum-based conditions of mESC cultivation allows to maintain a balance of mTOR/AMPK/Ulk1 pathways shifted to catabolic process to hold on cell pluripotency.

## Discussion

Despite the well-documented health benefits attributable to resveratrol, the intracellular mechanisms remain controversial because it can affect a variety of signaling pathways. One of the most robust and reproducible effects of resveratrol treatment is metabolic benefits in humans^21,22^. Resveratrol has been shown to mimic the metabolic effects of calorie restriction via activation of the AMPK/SIRT1 signaling^23^. Consistent with this, the accumulation of mESCs in the S phase of cell cycle might be a consequence of the tumor-suppressive effects conducted by the activated LKB1/AMPK pathway^24,25^. Formation of γH2AX foci is usually associated with cell cycle checkpoints^26,27^. According to our results, resveratrol treatment causes gradual accumulation of γH2AX foci and a decrease of proliferation in mESCs, evidencing for a metabolic stress that occurred in cells (Figs. 1 and 2). In many cells types a decreased metabolism leads to induction of autophagy to mitigate cell damage and provide nutrients for short-term survival^28^. According to the data obtained, mESCs treated with resveratrol do not undergo apoptotic cell death, and cells successfully overcome the emerging S-phase delay of cell cycle. These results are consistent with literature data that resveratrol promotes mESC survival^16,25^. Thus, S-phase delay may serve as a cellular protective mechanism for ESCs by triggering autophagy.

The complex containing Ulk1 is on the bifurcation point of autophagic process, so that autophagy is either activated by AMPK-mediated phosphorylation or suppressed by mTORC1 phosphorylation under conditions of nutrient starvation and/or growth factor deprivation. Our data provide evidence for resveratrol-induced autophagy going through mTORC1 repression  and AMPK/Ulk1 pathway activation. It is known that catabolic pathways play a role in the maintenance of pluripotency of ESCs^29,30^. According to our results, resveratrol increases expressions of pluripotency markers Oct3/4, Sox2, Nanog, Klf4 and a specific cell surface marker of undifferentiated state SSEA-1 in mESCs (Fig. 3). We assume resveratrol makes mESC population that is heterogeneous according to the degree of differentiation of individual cells more homogeneous. Therefore, the increase in the expression level in the whole population was caused by activation in that part of the cells that are in a less differentiated state. This is consistent with the result that more tightly packed colonies with mainly smooth boundaries are formed under resveratrol treatment probably due to the cadherin/β-catenin-mediated cell–cell junction up-regulation. The cadherin/β-catenin-mediated cell–cell junctions are known to play a pivotal role in providing compaction and blastocyst formation in the pre-implantation embryo^31^. Also, cytosolic β-catenin binds to the membranes and forms complexes with Oct3/4 and E-cadherin, which breaks down during differentiation^32^. Resveratrol treatment results in accumulation of β-catenin at the mESC membranes (Fig. 3).

There is little data uncovering resveratrol-dependent signaling pathways that regulate mESC pluripotency^16,33^. Here we show the mechanism of resveratrol action on pluripotency based on the mTORC1 pathway inhibition and autophagy activation via AMPK/Ulk1 (Figs. 4 and 5). Similar mechanisms are executed in blastocysts suspended in diapause in vivo and suspended blastocysts ex vivo exhibiting a marked decrease in mTOR pathway activity and autophagy activation^34^. The paused mESCs can be sustained in vitro for weeks without appreciable cell death. Since resveratrol-treated mESCs exhibit decreased proliferation without cell death preserving undifferentiated state, we suggest that resveratrol may have a similar effect on mESCs through mTOR inhibition and AMPK/Ulk1-dependent autophagy. Besides, transient inhibition of proliferation does not compromise self-renewal of mESCs^35^. The results obtained with Ulk1-overexpressed mESCs confirm the significance of AMPK/Ulk1 pathway activation for mESC pluripotency (Fig. 6). We showed that Ulk1 activation is accompanied by increased phosphorylation on Ser555 and Ser317 by AMPK. This is consistent with the latest data that activating phosphorylation of Ulk1 by AMPK on Ser555 is significantly higher in mESC compared to MEF^36^. The authors suggested that constitutive activation of Ulk1 by AMPK is an intrinsic signal pathway in ESCs to regulate their identity under normal physiological conditions. We aimed to clear this issue by generation of stable mESC line with doxycycline-inducible *ulk1* gene expression. In case of Ulk1 overexpression in DOX+ cells, AMPK does not slow down cell proliferation as compared with resveratrol-treated cells (data not shown). AMPK can exert growth-suppressive or growth-promoting effects depending on the cellular context^37^. For example, the AMPK activator AICAR increases mESC proliferation^24^. These results can be important for developing approaches to establish optimal conditions for mouse and human ESCs, especially slightly affecting the mTOR pathway. This is an useful point since the mTOR pathway inhibition induces differentiation in human pluripotent cells in vitro, while the commitment of mESCs to differentiation by LIF removal leads to the mTOR pathway upregulation^8,38^.

There is evidence that one of the biological effects of resveratrol on cells is the increase in the number of active mitochondria^10,39^. We also observed this effect in resveratrol-treated mESCs. The increase in the number of mitochondria and mitochondrial biogenesis is detected in cells undergoing differentiation^40^. However, no shift to differentiation was observed in resveratrol-treated cells (Fig. 3). We suggest that resveratrol induces mitochondrial rejuvenation that can be caused by autophagy induction, in particular mitophagy. This assumption is supported by the literature data demonstrating that resveratrol, acting through SIRT1, participates in the maintenance of mitochondrial homeostasis in the oocytes of cloven-hoofed animals by triggering mitophagy^41,42^. It has been shown that addition of resveratrol in the process of oocyte maturation induces mitochondria in the latter and then de novo synthesis that leads to the production of high-quality oocytes. Based on the data obtained, one may suggest a contribution of mitochondrial metabolism in resveratrol-treated cells to mESC pluripotency mediated through autophagy.

Summarizing, the autophagy, as the lysosomal pathway of protein degradation and cell reprogramming during cell growth, differentiation and cellular survival, is involved in a resveratrol-mediated beneficial effect on mESC pluripotency. Thus, mouse pluripotency can be captured in vitro by activating of the AMPK/Ulk1 signaling pathway that uncovers new opportunities for creating optimal conditions for mESC culturing with the following impact into human stem cell biology.

## Materials and methods

### Cell culture

Mouse embryonic stem cells IOUD2 were maintained on tissue culture dishes (Corning) coated with 0.2% porcine gelatin (Sigma) in a Dulbecco’s modified Eagle’s medium (DMEM)/F12 (1:1) (Biolot) supplemented with 0.1 mM 2-mercaptoethanol (Sigma), 10% fetal bovine serum (HyClone) and 1000 units/ml of murine recombinant LIF (Sigma) at 37 °C in an atmosphere of 5% CO_2_. HEK293T cells were maintained in DMEM (Biolot) supplemented with 10% fetal bovine serum (HyClone) at 37 °C in an atmosphere of 5% CO_2_. Resveratrol purchased from Calbiochem was added to the medium in a final concentration of 10 µM. Medium was changed daily. MRT68921 purchased from Selleckchem was used at a concentration of 2 µM

### Generation of the stable mESC line with doxycycline-inducible *ulk1* gene expression

For lentiviral packaging, HEK293T cells were seeded in a 10 cm dish 1 day before transfection. The indicated viral plasmids Lentidcas9-vp64-blast (Addgene #61425) or FgH1tUTG (Addgene #70183) were co-transfected with lentivirus packaging plasmids pMD2.G (Addgene #12259) and psPAX2 (Addgene #12260) in a 4:2:3 ratio using PEI MAX (molecular weight 40,000) (Polysciences, Inc., USA). The next day after transfection, media were changed to Opti-MEM® I Reduced Serum Media (Thermo Fisher Scientific). At 48 h after transfection, virus-containing media were collected by Amicon Ultra-15 Centrifugal Filter Units 100 kDa (Millipore), passed through a 0.45 µm polyethersulfone filter (Millipore), aliquoted and stored at −80 °C before infection or titration. mESCs were seeded 1 day before transduction. The next day, the medium was changed to fresh and protamine sulfate 50 μg/ml (Sigma) and Lentidcas9-vp64-blast or FgH1tUTG were added. Infected green fluorescent protein-positive cells mESCs were sorted in BD FACSAria™ III. FgH1tUTG-transduced cells were infected with Lentidcas9-vp64-blast and selected by 5 µg/ml of blasticidin (Sigma) for 1 week. Oligonucleotides were designed using the online tools http://sam.genome-engineering.org/database and https://www.nature.com/articles/nbt.2647: F-TCCCGAGGCGGGGCGGGGCTTAGTT; R-AAACAACTAAGCCCCGCCCCGCCTC; and cloned into FgH1tUTG vector.

### Fluorescence-activated cell sorting (FACS) analysis of cell cycle distribution

For cytometric analysis of DNA content, cells were harvested, washed with phosphate-buffered saline (PBS) and incubated for 30 min at room temperature in PBS containing 0.01% of saponin (Sigma). Cells were washed twice with PBS and incubated with 100 µg/ml RNase A and propidium iodide (PI) for 15 min at 37 °C.

Cytometric assessment of SSEA-1, LC3, Oct3/4 and Nanog proteins was carried out according to the previously described protocol^43^. Briefly, cell suspension was washed with PBS and fixed with ice-cold 1% methanol-free formaldehyde solution in PBS. Then, cells were pelleted, washed twice with PBS and were stored in ice-cold 70% ethanol at −20 °C for at least 12 h. Cells were incubated with antibodies against SSEA-1 (Invitrogen #41–1200), LC3 (MBL #PM036), Oct3/4 (#sc-5279) and Nanog (#sc-376915) (Santa Cruz) overnight at 4 ºC. After that, samples were incubated with secondary antibodies AlexaFlour-568/633-conjugated goat anti-mouse/rabbit F(ab′)2 fragment (Invitrogen) for 1 h at room temperature (RT), then treated with RNAse (1 mg/ml, 30 min, RT) and 5 µl of 1 mg/ml of PI was added.

Cells were stained for 30 min with MitoTracker red (Invitrogen #M7512), MitoTracker green (Invitrogen #M7514) and LisoTracker green (Thermo Fisher Scientific, #L7526) in growth media at a concentration of 100 nM in a 37 °C 5% CO_2_ culture incubator. Cells were washed twice in PBS and immediately analyzed using flow cytometer.

Flow cytometric analysis of Cyto-ID Green Detection Reagent stained cells was performed according to the manufacturer’s protocol (Autophagy Detection Kit, ENZ-51031-K200, Enzo Life Sciences).

Annexin V staining was carried out with the Annexin V-FITC Apoptosis detection Kit (Abcam, #ab14085) according to the manufacturer’s protocol.

Samples were analyzed using a flow cytometer CytoFLEX (Beckman Coulter) with 405 nm, 488 nm and 638 nm lasers. Cell cycle phase distribution analysis was performed with MODFIT LT 3.0 software (Verity Software House). A minimum of 10,000 cells were analyzed for each sample.

### Immunofluorescence

Cells were seeded and grown on gelatine-coated coverslips, rinsed with cold PBS, fixed in 4% paraformaldehyde for 20 min at RT and permeabilized with 0.25% Triton X-100 for 25 min. After three washes cells were incubated in blocking solution (5% bovine serum albumin (BSA) from Sigma in PBS) and then incubated with primary antibodies against β-catenin (Santa Cruz) at 40 C overnight. After washing, samples were incubated for 1 h with secondary antibodies AlexaFlour-568-conjugated goat anti-mouse F(ab′)2 fragment (Invitrogen). Primary and secondary antibodies were dissolved in PBS with 5% BSA and 0.1% Tween-20 (Sigma). Nuclei were stained by 5 min of incubation with 4′,6-diamidino-2-phenylindole (DAPI). Images were analyzed with the confocal microscope Olympus FV3000.

### Protein lysate preparation and western blotting

For immunoblotting, cell lysates were obtained by incubating cells in RIPA buffer containing PBS solution, 1% Igepal, 0.5% sodium deoxycholate, 0.1% SDS (Sigma), protease and phosphatase inhibitors (cocktail Complete, Roche), 5 mM EGTA and 10 mM β-glycerophosphate. Equal amounts of protein extracts were run on polyacrylamide gel electrophoresis, transferred to PVDF-FL membranes (Millipore) and blotted with primary antibodies according to the manufacturer’s recommendations. Horseradish peroxidase-conjugated goat anti-rabbit and rabbit anti-mouse antibodies (Pierce) were used as secondary antibodies. Proteins on membranes were visualized by means of ECL (Amersham). The relative band intensity (normalized to α-tubulin) was quantitated using Image Lab version 6.0 (Bio-Rad).

Primary antibodies were as follows: Oct3/4 (#sc-5279), Sox2 (#sc-17320) and Nanog (#sc-376915) from Santa Cruz Biotechnology; α-tubulin (Sigma #T5168); GAPDH (#2118), H2AX (Ser139) (#9718), Klf4 (#4038), Atg5/Atg12 (#8540), Beclin-1 (#3738), AMPK (#5832), AMPK Thr172 (#2535), Ulk1 (#8054), ULk1 Ser555 (#5869), Ulk1 Ser757 (#14202), Ulk1 Ser317 (#12753), mTOR Ser2448 (#5536), pS6 Ser235/236 (#2211), p4EBP1 Thr37/46 (#2855), p70S6K Thr389 (#9206) (Cell Signaling); LC3 (#PM036) from MBL International; p62 (#610832) from BD Biosciences-US.

### qRT-PCR

For qRT-PCR, total cellular RNA was isolated using TRIzol® (Invitrogen) according to the manufacturer’s protocol. Reverse transcription was performed with 2 µg RNA, using random hexaprimers (Promega) and M-MuLV Revertase (RevertAid, Fermentas). qPCR was performed using the Real-Time PCR Reagent kit with SYBR Green dye and the reference dye ROX (Syntol), on the 7500 Real-Time PCR System (Applied Biosystems). The reaction parameters were according to the manufacturer's instructions (5 min at 95 °C, then 60 °C for 50 s and 95 °C for 15 s repeated in 45 cycles). The following gene-specific primers were used: oct-3/4-F, CAAGTTGGCGTGGAGACT; oct-3/4-R, TTCATGTCCTGGGACTCCTC; nanog-F, GATGCAAGAACTCTCCTCCA; nanog-R, CAATGGATGCTGGGATACTC; sox2-F, -ACATGAACGGCTGGAGCAACG, sox2-R, CATGTAGGTCTGCGAGCTGGTC; klf4-F, GACTAACCGTTGGCGTGAG, klf4-R; CGGGTTGTTACTGCTGCAAG, prdm14-F, GCCTGAGATCCGCAAAACT, prdm14-R, CATGCGCGTAGTAGGTAGTC; esrrb-F, TAGGGGTTGAGCAGGACAAG, esrrb-R, CTACCAGGCGAGAGTGTTCC; GGAGCCGTGACCACTGACA, ppargc1a-R, TGGTTTGCTGCATGGTTCTG; atg5-F, CACTGGGACTTCTGCTCCTG, atg5-R, CAACCAAAGCCAAACCGAGG; atg12-F, TAAACTGGTGGCCTCGGAAC, atg12-R, CCATCACTGCCAAAACACTCA; lc3-F, CCGACCGGCCTTTCAAGC, lc3-R, CGATGATCACCGGGATCTTACTGG; mtor-F, CAAGATGCTTGGGACGGGT, mtor-R, CATTCCGGCTCTTCAGTCCA; β-actin-F, CCGTAAAGACCTCTATGCCAAC, β-actin-R, ATGGAGCCACCGATCCACA. The data were normalized to *β-actin*  mRNA levels. SEM was calculated for 4 technical replicates within the experiment.

### Alkaline phosphatase staining

Alkaline phosphatase staining was carried out with the leukocyte alkaline phosphatase kit (Sigma, #86R) according to the manufacturer’s protocol.

### Enzyme activity assay

LDH and aldolase activity in cells was measured by the LDH Activity Assay Kit (Sigma-Aldrich, #MAK066) and Aldolase Activity Assay Kit (Sigma-Aldrich, #MAK233) according to the manufacturer's instructions. In these assays, LDH and aldolase reduce NAD to NADH, which is specifically detected by colorimetric (450 nm) assay. Total protein concentration of cell extract was determined by the Bradford method after the use for enzyme activity assays and the values obtained from specific enzyme activities were normalized by the total protein concentration in cell extract.

### xCELLigence real-time cell analysis

The proliferation analysis was performed using xCELLigence RTCA DP system (ACEA Biosciences, CA, USA) according to the manufacturer’s instructions with minor modifications. The xCELLigence RTCA DP system provides a platform for label-free and operator-independent investigation of cells in physiologically relevant conditions. For cell proliferation assays, 1 × 10^3^ cells were seeded in each well of ACEA E-plate 16 in medium. Cell index was registered every 10 min for 60 h. Each experiment was performed at least in 4 replicas.

### Statistical analysis

Statistical analyses were conducted using GraphPad 5.0 software. Student’s *t*-test was used for comparisons. Data were presented as the mean ± relative standard deviation (rSD) or standard error of the mean (SEM); *p < 0.05, **p < 0.001, ***p < 0.0001.
